# Exploring Morphologic and Functional Variants in Hypertrophic Cardiomyopathy: An Echocardiographic and Doppler Review

**DOI:** 10.3390/diagnostics15212688

**Published:** 2025-10-24

**Authors:** Kamil Stankowski, Fabrizio Celeste, Manuela Muratori, Francesco Cannata, Nicola Cosentino, Fabio Fazzari, Laura Fusini, Daniele Junod, Massimo Mapelli, Riccardo Maragna, Andrea Baggiano, Saima Mushtaq, Luigi Tassetti, Gianluca Pontone, Mauro Pepi

**Affiliations:** 1 Department of Perioperative Cardiology and Cardiovascular Imaging, Centro Cardiologico Monzino IRCCS, Via C. Parea 4, 20138 Milan, Italy; fabrizio.celeste@ccfm.it (F.C.); nicola.cosentino@ccfm.it (N.C.); laura.fusini@cardiologicomonzino.it (L.F.); andrea.baggiano@ccfm.it (A.B.); gianluca.pontone@ccfm.it (G.P.);; 2 Department of Critical Cardiology and Rehabilitation, Centro Cardiologico Monzino IRCCS, Via C. Parea, 4, 20138 Milan, Italy; massimo.mapelli@ccfm.it; 3 Department of Clinical Sciences and Community Health, University of Milan, Via Parea, 4, 20138 Milan, Italy; 4 Department of Biomedical, Surgical and Dental Sciences, University of Milan, Via Festa del Perdono 7, 20122 Milan, Italy

**Keywords:** hypertrophic cardiomyopathy, echocardiography, left ventricular outflow gradient, left ventricular apical aneurysm, mitral valve, systolic anterior movement

## Abstract

Hypertrophic cardiomyopathy (HCM) is a complex and heterogeneous myocardial disorder, best evaluated with echocardiography for initial diagnosis, risk stratification, and longitudinal monitoring. This focused review explores the echocardiographic assessment of various morphologic phenotypes of HCM, emphasizing their diagnostic nuances. Distinct phenotypes, including asymmetric septal hypertrophy, concentric hypertrophy, and the less common apical HCM, present unique imaging challenges. Additionally, the review outlines essential techniques and practical tips for assessing left ventricular apical aneurysm flow patterns and dynamic intraventricular gradients. A thorough understanding of mitral valve anatomy and its role in left ventricular outflow tract obstruction is also crucial. Finally, anatomical variants of the mitral valve, papillary muscles and left ventricular myocardium are examined for their contribution to systolic anterior motion and mid-ventricular obstruction as well as for constituting additional phenotypical expressions of HCM, beyond left ventricular hypertrophy.

## 1. Introduction

Echocardiography is central to the diagnosis and monitoring of patients with hypertrophic cardiomyopathy (HCM). Echocardiographic evaluation underpinning obstructive and non-obstructive HCM mainly consist of the description of the magnitude and distribution of left ventricular (LV) hypertrophy—whether symmetrical or asymmetrical (e.g., apical, septal or of other myocardial segments), the presence of left ventricular outflow tract (LVOT) obstruction, and diastolic dysfunction. The data derived from echocardiography are then integrated with clinical data, exercise evaluation, genetic testing, and information obtained from cardiac magnetic resonance (CMR), especially in cases with conflicting diagnostic information, which may suggest potential phenocopies or influence risk assessment [1,2,3,4,5,6,7].

However, several other anatomical and functional anomalies may be present and a more comprehensive echocardiographic and Doppler evaluation may further refine the diagnosis, prognosis and therapy. Moreover, Doppler evaluation of LVOT or intraventricular signals and mitral regurgitation can be challenging to interpret. This has assumed greater importance in the current era since the introduction of new medical and surgical therapies for treating obstructive HCM [8,9]. While echocardiography remains the first-line imaging modality for the assessment of HCM due to its accessibility, real-time hemodynamic assessment, and widespread familiarity among clinicians, CMR serves as an invaluable complementary tool, given its additive value in diagnosis, risk stratification, and tissue characterization. The integration of both modalities often provides a more complete diagnostic and risk stratification framework in HCM.

The present review discusses how a detailed echocardiographic evaluation may better define not only typical findings, but also more complex conditions and anatomical variants. While numerous reviews have comprehensively addressed HCM, this manuscript aims to provide a uniquely practical perspective by focusing on Doppler-based diagnostic nuances and real-world ‘tips and tricks’ that are directly applicable in clinical practice. Additionally, we dedicate particular attention to less commonly discussed morphologic phenotypes, such as apical HCM with and without aneurysms, as well as intraventricular gradients, mitral and LV anatomical variants.

## 2. Distribution of Segmental Hypertrophy

In HCM, the distribution of hypertrophy can vary, but the most common pattern is asymmetrical septal hypertrophy with involvement of the anterior basal or mid septum and the adjacent anterior wall [10,11,12,13,14]. When septal hypertrophy predominates, the commonly used cutoff for maximal wall thickness in HCM is 15 mm, with a septal-to-posterior wall ratio of 1.3:1 or greater. In systemic hypertension, a septal-to-posterior wall thickness ratio > 1.5:1 is recommended to define HCM [15]. In family members of a HCM proband, an unexplained septal thickness of 13 mm may be used to suggest disease presence, particularly when the electrocardiogram reading is abnormal. When a pathogenic, disease-causing mutation is detected, the diagnosis is established; however, echocardiography may be normal in early stages of the disease and even minimal hypertrophy may reflect clinical disease onset [16]. Beyond fixed thickness thresholds, Shiwani et al. recently suggested that a demographic-adjusted approach to LV hypertrophy assessment, using personalized thresholds based on age, sex, and body size, may improve diagnostic accuracy, reducing overdiagnosis in elderly hypertensive patients and increase sensitivity in young women [17].

**Figure 1:**
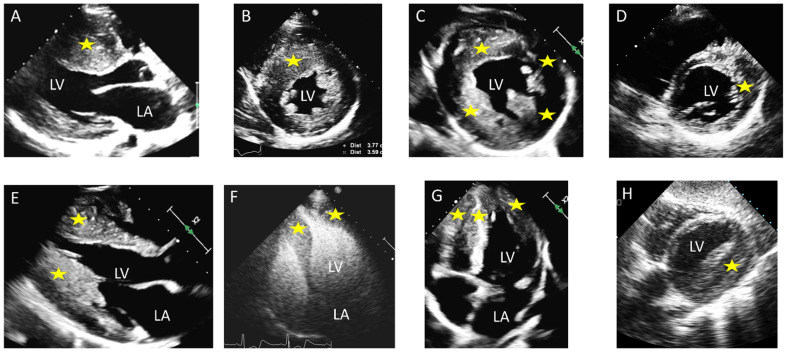
Different distribution and location of hypertrophy in hypertrophic cardiomyopathy (HCM) (asterisks): (**A**) (parasternal long axis) and (**B**) (parasternal short axis) septal hypertrophy; (**C**) (parasternal short axis) concentric hypertrophy; (**D**) (parasternal short axis) lateral hypertrophy; (**E**) (parasternal long axis) and (**F**) (contrast echocardiography, apical four-chamber) apical hypertrophy; (**G**) (apical four-chamber) left ventricular (LV) and right ventricular apical hypertrophy; (**H**) (subcostal view) lateral hypertrophy. LA: left atrium.

Akin to disproportionate septal thickening, loss of apical tapering is another hallmark of HCM. The normal LV thickness progressively reduces from the base toward the apex. In some patients with HCM there is abnormal thickening of the apical myocardium, which leads to a blunted or rounded apex (i.e., reduced apical angle), and loss of the usual tapering [18], most evident on contrast-enhanced echocardiography or CMR. Therefore, societal guidelines highlight that in patients with known or suspected HCM it is essential that all LV segments from base to apex be examined, ensuring that the wall thickness is recorded at basal, mid and apical levels of the LV [1]. Examples of typical and more uncommon HCM phenotypes with different hypertrophic patterns are shown in Figure 1.

In terms of LV hypertrophy distribution, septal hypertrophy with asymmetric hypertrophy (septal-to-posterior wall ratio > 1.3/1.5) represents about 55% of HCM, concentric hypertrophy 31% and apical hypertrophy 14% [19]. The prevalence of the different distribution patterns of hypertrophy has been confirmed by CMR (Table 1), an exam that may overcome limitations of echocardiography in suboptimal examinations [12,20,21].

The degree and location of myocardial hypertrophy in HCM are highly variable and carry significant prognostic implications, necessitating precise characterization. Table 2 summarizes the diagnostic, hemodynamic and clinical characteristics of the various forms of HCM.

The hypertrophy pattern also serves as a predictor of genotype positivity. Specifically, patients exhibiting a reverse curvature or neutral septal morphology have approximately a 50% likelihood of harbouring a pathogenic mutation, whereas those with focal basal hypertrophy or apical HCM show a diagnostic yield of <20%. Younger individuals with HCM more frequently present with the reverse curvature pattern, while the sigmoid septal morphology is predominantly observed in elderly patients, necessitating differentiation from a nonhereditary form of hypertrophy [22]. Furthermore, a recent paper by Maurizi et al. demonstrated that the identification of severe posterior LV wall hypertrophy (i.e., >15 mm) at echocardiography was almost exclusively observed in non-sarcomeric hypertrophic phenotypes [23].

HCM patients sometimes present with right ventricular hypertrophy [24,25]. Maron et al. identified right ventricular hypertrophy, characterized by a wall thickness exceeding 8 mm, in 15 of 46 patients with HCM (33%), with 4 patients (9%) exhibiting thickness greater than 10 mm [24]. Generally, obstruction of the right ventricular outflow tract is a rare finding in adult HCM patients. Shimizu et al. reported that right ventricular outflow tract obstruction, defined as a flow velocity ≥ 2 m/s, was found in 15% of HCM patients [26].

## 3. Apical Hypertrophy (With or Without Aneurysm)

Also known as “Yamaguchi syndrome”, apical HCM was once considered almost exclusive to the Japanese population but is now recognized in diverse ethnic groups worldwide. Apical HCM represents about 25% of HCM cases in Asian populations, compared to a prevalence of 1–10% in non-Asian groups, and has a relatively benign prognosis in terms of cardiovascular mortality [18,27,28,29,30]. On the contrary, HCM with apical aneurysm is associated with substantial cardiovascular morbidity and mortality [31,32]. Indeed, patients with HCM who develop LV apical aneurysms face an increased risk of thromboembolic complications and arrhythmic sudden cardiac death. Recognizing this phenotype enhances risk stratification and enables appropriate treatment strategies to address such potentially life-threatening complications [29,32,33].

Approximately 2–10% of patients with apical HCM have been reported to exhibit apical regional dysfunction, outpouching, or an aneurysm in the presence of normal coronary arteries. Yang et al. examined a large population of HCM and found aneurysms in 31 out of 1332 apical HCM patients (2.3%) [34]. Sherrid demonstrated that the vast majority (95%) of patients with apical aneurysms also have concomitant mid-ventricular obstruction [35].

**Figure 2:**
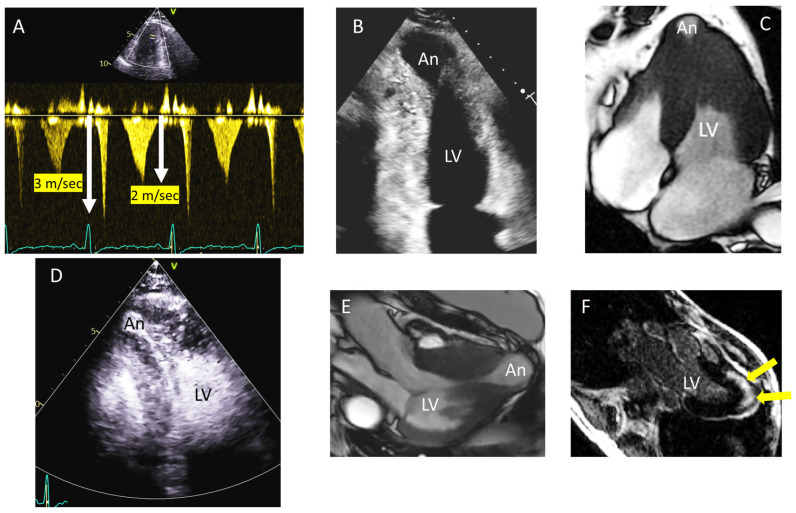
Apical HCM with aneurysm. Typical Doppler patterns, contrast echocardiography and cardiac magnetic resonance (CMR) aid in the detection of apical aneurysms. (**A**) A typical continuous Doppler void pattern (early systolic high velocity towards the LV cavity—3.3 m/s, and early diastolic paradoxical flow—2.2 m/s, known as the ‘lobster claw’ abnormality). (**B**) Two chamber view with a clear detection of the aneurysm (An). (**C**) CMR (cine four-chamber view) showing a small aneurysm and (**D**) same case as demonstrated by contrast echocardiography. (**E**) CMR (cine three-chamber view) of a larger aneurysm. (**F**) CMR (late gadolinium enhancement three-chamber view sequence) showing transmural apical scarring (arrows). An: aneurysm.

In a recent paper we tried to define whether there is a typical Doppler pattern at the level of obstruction in patients with apical HCM with aneurysm [36]. Doppler flow patterns help clarify the disease pathophysiology: the paradoxical diastolic flow jet moving from the apex toward the base suggests elevated intracavitary pressure in the LV apex, which in turn leads to reduced coronary perfusion pressure. Obstruction of the LV cavity may lead to the cessation of systolic flow at the mid-ventricular level, leading to blood being trapped in the apical chamber. Consequently, despite normal coronary arteries, ischemia and finally necrosis may occur leading to the typical apical aneurysms (Figure 2) [37].

Three different flow patterns at the mid-ventricular level using continuous wave Doppler have been identified. The most frequent pattern displayed early systolic flow from the LV apex to the base, interrupted during systole (void) and followed by early diastolic emptying (also known as the ‘lobster claw’ abnormality). A second pattern was identified as blunted early systolic flow and paradoxical early diastolic flow, while in only one case out of 19 a paradoxical flow both in systole and diastole was registered [36].

**Figure 3:**
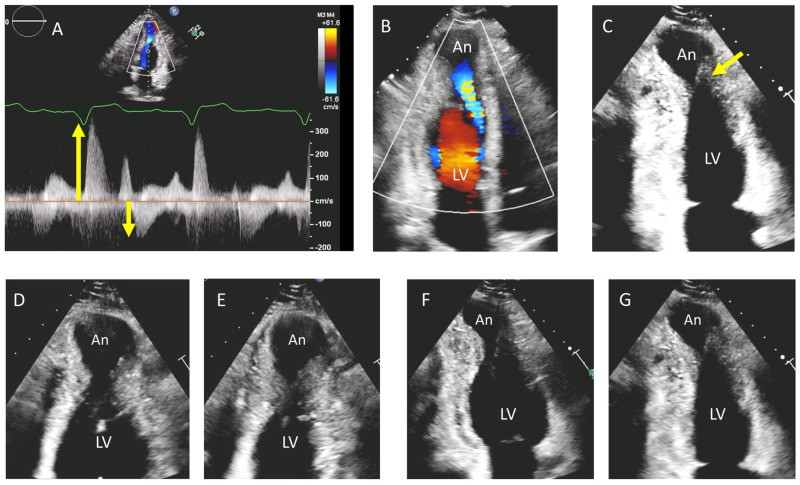
Apical HCM with aneurysm. Modified apical views may be necessary to identify the site of mid-ventricular LV obstruction and confirm the systolic expansion of the apical aneurysm. In this case the Doppler (**A**) demonstrates an early systolic and early diastolic paradoxical flow (arrows). (**B**) The colour Doppler facilitates the identification of the mid-ventricular LV obstruction. (**C**) An adapted two-chamber view shows the obstruction (arrow) and the apical aneurysm (An). Diastolic (**D**) and systolic (**E**) frames of the same case (four-chamber view) and diastolic (**F**) and systolic (**G**) frames (two-chamber view) demonstrating expansion of the apical cavity in systole.

Figure 3 shows an apical HCM case with clear paradoxical flow in early systole and early diastole associated with severe mid-apical LV obstruction and a dyskinetic apical aneurysm. In the classical void pattern, severe obstruction combined with apical dysfunction cause a virtual absence of flow at the site of obstructing neck: this explains the mid-systolic Doppler signal void. Paradoxical early diastolic flow is later caused by the high pressure into the apical cavity that empties into the LV during relaxation (early diastolic phase).

There is a spectrum of aneurysm size from very small to large and the echocardiographic evaluation is not always straightforward, particularly in smaller ones. A colour Doppler signal at the neck of the cavity may suggest obstruction and off-axis views focused to the apical near field may facilitate the recognition of the cavity. In these cases, both the Doppler patterns (colour, pulsed and continuous wave) as well as contrast agents for aneurysm detection may precede CMR. Lee et al. showed that contrast echocardiography has high sensitivity for detecting LV apical aneurysms and should be used routinely in the evaluation and risk stratification of patients with HCM. Contrast echocardiography demonstrated a sensitivity of 98%, significantly higher than the 67% sensitivity observed with non-contrast echocardiography (equivalent detection as by CMR) [38]. However, CMR not only may confirm the diagnosis and precisely describe the morphology of the LV cavity and mid-ventricular obstruction but may also define the presence and extension of fibrosis (and its transmurality), if present, which may be the key driver of adverse arrhythmic outcomes [37,39]. The presence of an aneurysm may have clinical implications in terms of anticoagulation and/or ventricular arrhythmias ablation [32]. Also, as these patients are at higher risk of sudden cardiac death, placement of an implantable cardioverter-defibrillator may be warranted [29].

## 4. Tip and Tricks Concerning LVOT Obstruction Doppler Measurement

HCM is frequently an obstructive disease, with ~70% of patients having mechanical impedance to LV outflow (defined as a maximum gradient ≥ 30 mmHg) at rest or with physiological provocation (i.e., Valsalva manoeuvre, exercise) [40,41,42,43]. Although the LVOT gradient is not so clearly correlated with functional capacity or symptoms in obstructive HCM, its assessment remains a key objective of echocardiography [44].

Recognizing the various obstructive HCM phenotypes (dynamic LVOT obstruction, mid-ventricular obstruction, combined obstruction) as well as the anatomical abnormalities that predispose to obstruction has management implications [45]. In many cases the typical dagger shape of the LVOT gradient facilitates the accurate measurement of the maximal gradient both at rest or after Valsalva or exercise. However, in some cases, the morphology of the Doppler velocity envelope is atypical, or the presence of extremely high velocities complicates interpretation. Challenges may also arise when the Doppler signal from LVOT obstruction overlaps with the mitral regurgitation jet; in such cases, even with adjustments in probe positioning, distinguishing between the two signals can be difficult.

**Figure 4:**
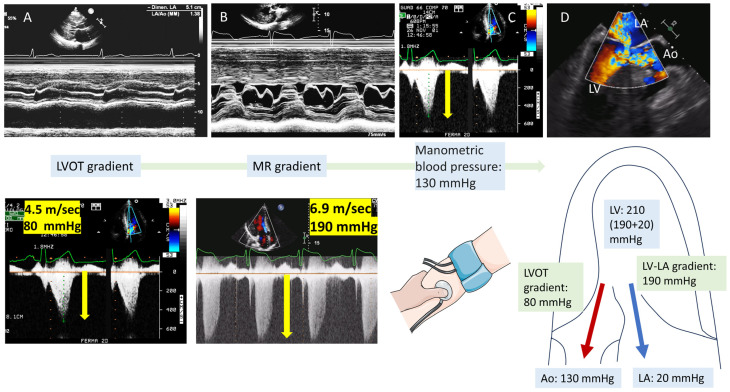
(**A**) Mid-systolic closure of the aortic box. (**B**) M-mode of the mitral valve with systolic anterior movement (SAM). (**C**) Left ventricular outflow tract (LVOT) obstruction with a typical dagger shape morphology. (**D**) Three-chamber long axis transesophageal echocardiography (TEE) view; the colour Doppler demonstrates the LVOT high velocity and the coexistence of mitral regurgitation (MR) due to SAM. Lower panels: how to calculate and validate the correct LVOT gradient from the MR gradient and manometric blood pressure (scheme of the simple test that may confirm the accuracy of the LVOT gradient measurement). Because the MR gradient reflects the difference between LV systolic pressure and left atrial pressure, it can be used to estimate LV systolic pressure when left atrial pressure is known or assumed. Simultaneously, the cuff-measured systolic blood pressure reflects aortic pressure, provided there is no significant aortic stenosis or LVOT obstruction. In HCM, the difference between the estimated LV systolic pressure and systolic blood pressure should equal the Doppler-derived LVOT gradient. If it does not, the LVOT gradient may be inaccurate, commonly underestimated due to poor Doppler alignment, or overestimated if the signal is contaminated by the MR jet. Ao: aorta.

In Figure 4 we show a simple method to avoid overestimation of the LVOT gradient by measurement of the systolic blood pressure, the supposed LVOT gradient and the LV-left atrium (LA) gradient, i.e., the mitral regurgitation maximum velocity.

As shown, measurements of the LVOT gradient (the measurement to be verified), LV-LA gradient and manometric systolic blood pressure allow to determine and identify the congruence of the LVOT gradient. The systolic LV pressure is given by the sum of the LV-LA gradient and the LA systolic pressure (by convention ~20 mmHg), similarly to the pulmonary pressure formula that utilizes the right ventricle-right atrium gradient plus the right atrial pressure [46]. The correct LVOT gradient should approximate the difference between the LV pressure taking away the manometric blood pressure.

Another technically difficult condition is the presence of asymmetric basal septal hypertrophy in the setting of valvular aortic stenosis. In these cases, the differential diagnosis (HCM associated with aortic stenosis or severe aortic obstruction leading to disproportionate septal hypertrophy) is even more challenging and based mainly on the anatomy of the aortic valve. It is also difficult to separate these two flow patterns and to define which is the main component of the pressure gradient. Moreover, in the presence of two obstructions in series, the correct application of the continuity equation is not possible. Pulsed Doppler (with the high pulse repetition frequency and the sample volume in the LVOT) and/or superimposition of the two patterns by continuous Doppler (LVOT obstruction with the dagger shape pattern and aortic flow with a holosystolic flow pattern) may partially overcome these difficulties and facilitate decisions regarding therapies (such as myomectomy associated with aortic valve replacement). Also, strain imaging assists in differential diagnosis by showing regional strain reduction localized to hypertrophied segments (e.g., basal septum or apex) in HCM, whereas aortic stenosis causes a more global, symmetrical reduction in longitudinal strain, particularly in basal segments. Dobutamine stress echocardiography in HCM often provokes or increases a dynamic LVOT gradient, unmasking latent obstruction, whereas aortic stenosis represents a fixed valvular obstruction.

Similarly, in elderly patients, aortic stenosis overlapping with transthyretin amyloid cardiomyopathy should be considered in the differential diagnosis, particularly when there is disproportionate LV wall thickening relative to the degree of pressure overload [47,48].

## 5. Mitral Valve Anomalies

Groarke et al. showed that sarcomere gene mutations in HCM are associated with primary abnormalities of the mitral valve (MV), such as elongation of the anterior leaflet relative to the size of the LV cavity and anterior displacement of the papillary muscles, anatomic features that promote systolic anterior motion (SAM) of the anterior mitral leaflet [49].

Sherrid et al. described several MV anomalies in detail. Most patients with obstructive HCM have elongated anterior and posterior mitral leaflets, as compared with normal subjects. In particular, the anterior mitral leaflet in HCM typically measures around 34 mm, compared to an average of 24 mm in normal hearts. Elongated leaflets extend into the LV cavity, projecting an average of 26 mm above the mitral annular plane—approximately double the 13 mm seen in normal hearts [50]. Consequently, the protruding mitral leaflet plays an important role in the pathophysiology of SAM, and may reduce the efficacy of isolated septal reduction, necessitating concomitant MV intervention.

It has also been showed that in patients with obstructive HCM secondary chords are often thickened and exert a tethering force on the anterior mitral leaflet, drawing it toward the septum and thereby contributing to SAM.

Moreover, the papillary muscles in HCM frequently exhibit two key anomalies: anterior displacement of the base of the anterolateral papillary muscle and/or an abnormal muscular connection between its head and the anterolateral LV wall, typically inserting into or near the lateral scallop of the anterior leaflet (A1). A higher frequency of bifid papillary muscles has also been described [50]. Severity of obstruction correlated with these anomalies independently of septal thickness.

In other cases, insertion of a head of the anterolateral papillary muscle directly into the middle of the anterior leaflet without intervening chordae has been described. Finally, rarely calcification of the mitral leaflets or annulus occurs in patients with SAM and leaflet-septal contact.

**Figure 5:**
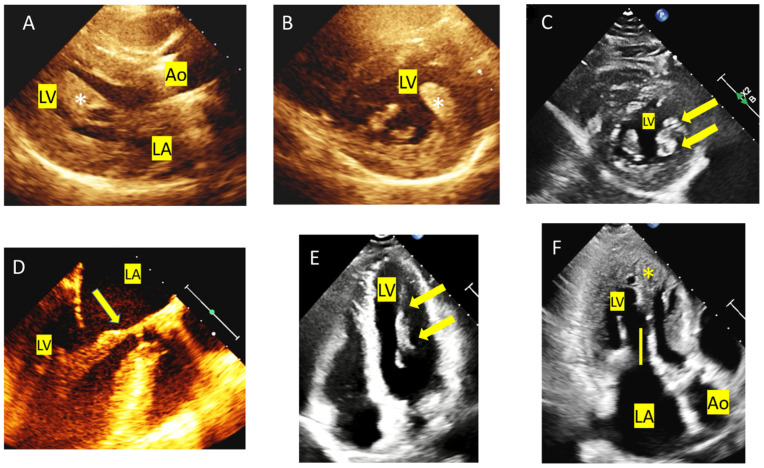
Anomalies of the mitral valve (MV) apparatus. (**A**) Parasternal long axis view showing an anteriorly displaced and hypertrophied papillary muscle (asterisk). (**B**,**C**) Parasternal short axes of the LV: a hypertrophic anterolateral papillary muscle (**B**, asterisk) and a bifid one (**C**, arrows). (**D**) TEE showing the insertion of a head of the anterolateral papillary muscle directly into the mid portion of the anterior mitral valve leaflet (arrow). (**E**) Four-chamber apical view showing an abnormal apical insertion of the papillary muscle that is also anteriorly displaced (arrows). (**F**) Three-chamber apical view that allows the measurement of a long anterior mitral leaflet (yellow bar); asterisk showing the anterolateral papillary muscle.

Table 3 lists the main anomalies of the MV apparatus and Figure 5 shows several examples of MV anomalies in HCM.

## 6. Left Ventricular Anomalies

New CMR data have been recently published revealing a more complex pattern of LV remodeling in HCM, characterized not only by basal septal hypertrophy but also by LV lengthening, apical dilatation, and inward remodeling of the LVOT [51]. A hypertrophied basal anteroseptal wall, along with inward remodeling of this region, plays a key role in reducing the LVOT area and amplifying Venturi and drag forces, both of which contribute to the development of dynamic obstruction. The reduced size and altered geometry of the LVOT decrease the space between the septum and the elongated MV leaflets, increasing the likelihood of leaflet-septum contact. Transferring these CMR data to echocardiography, three-dimensional echocardiography may be used to assess the LVOT in terms of dimensions and geometry.

An accessory LV apical-basal muscular bundle is a common anomaly in HCM. This muscle bundle stretches from the LV apex to the basal septum or anterior wall, traversing the LV cavity.

Another LV anomaly described mainly at CMR, sometimes visible at echocardiography, is the presence of crypts, i.e., narrow, contractile, blood-filled invaginations within the LV myocardium. The definition of myocardial crypts is not yet entirely settled, making comparisons of prevalence between studies difficult. Single or multiple crypts or clefts have been associated with HCM [52], but whether they represent pre-phenotypic markers of the disease, or benign anatomical variants remains a subject of debate. Overall, crypts are found in ~5–15% of HCM patients, though prevalence varies by modality [53].

Maron et al. showed that myocardial crypts represent a unique structural feature of HCM, occurring frequently (i.e., 61%) in genotype positive-phenotype negative subjects, rarely (i.e., 4%) in subjects who had already developed LV hypertrophy, and being absent in control subjects [54]. In the same study, thorough examination of two-dimensional echocardiograms failed to detect myocardial crypts in any of the patients. Spacek first reported a case of myocardial crypt in a single subject at echocardiography [55], but large studies are lacking. Future prospective studies should clarify whether crypts predict genotype-positive HCM in otherwise normal LV morphology.

Finally, microvascular dysfunction represents a key pathophysiological feature of HCM, arising from structural and functional alterations in the coronary microcirculation, including arteriolar remodeling, reduced capillary density, and increased extravascular compression. Despite the absence of epicardial coronary artery disease, these abnormalities result in impaired myocardial perfusion. Doppler echocardiography enables its non-invasive evaluation through measurement of coronary flow reserve, which is typically reduced. Assessment is commonly performed in the distal left anterior descending artery, where diastolic flow velocities are often elevated relative to normal controls, reflecting increased microvascular resistance and impaired vasodilatory capacity [56,57].

## 7. Conclusions

The diagnosis of HCM primarily relies on the identification of increased LV wall thickness, however the phenotypic spectrum of HCM extends beyond hypertrophy, encompassing intraventricular obstructive gradients, distinct morphologic patterns, abnormalities of the MV apparatus and/or LV myocardium. Comprehensive imaging evaluation is essential for accurately characterizing these features, which are critical for guiding clinical management. The emergence of novel therapies such as cardiac myosin inhibitors alongside established pharmacologic, interventional, and surgical treatments, renders detailed imaging even more pivotal, especially in patients with atypical presentations. With new therapies such as mavacamten and aficamten, precise imaging characterization will directly guide therapeutic eligibility. In this context, echocardiography remains the cornerstone of initial diagnostic assessment. When meticulously performed, it provides invaluable structural and hemodynamic information, facilitating integration with clinical findings and complementary imaging modalities, particularly CMR in diagnostically complex cases. Finally, strain imaging and AI-augmented echocardiography will increasingly enhance diagnostic accuracy in the future.

## Figures and Tables

**Table 1:** Left ventricular hypertrophy distribution as assessed by transthoracic echocardiography and cardiac magnetic resonance in published literature.

Left Ventricular Hypertrophy Distribution		
	Transthoracic Echocardiography (Adapted from Shapiro et al. [19])	Cardiac Magnetic Resonance (Adapted from Noureldin et al. [20])
Septal	55%	~66%
Concentric	31%	5–24%
Apical	14%	<10%

**Table 2:** Diagnostic, hemodynamic and clinical characteristics of the various forms of hypertrophic cardiomyopathy. CMR: cardiac magnetic resonance; ECG: electrocardiogram; HCM: hypertrophic cardiomyopathy; LGE: late gadolinium enhancement; LV: left ventricular; LVH: left ventricular hypertrophy; LVOT: left ventricular outflow tract; RV: right ventricular; SAM: systolic anterior motion; SCD: sudden cardiac death.

HCM Type	Septal (Asymmetric HCM)	Concentric HCM	Apical HCM
Definition/Morphology	Asymmetric septal hypertrophy, typically septum > 1.3 times thicker than posterior wall	Symmetric thickening of all LV walls	Predominantly apical hypertrophy of LV
Epidemiology	Most common form of HCM	Overlap with hypertensive heart disease	More common in East Asian populations
ECG Findings	LVH, deep Q waves in inferior/lateral leads	LVH, diffuse ST-T changes	Deep negative T waves (especially in precordial leads), LVH
Echocardiography	Septal hypertrophy; LVOT obstruction; SAM of the anterior mitral leaflet ± SAM-associated mitral regurgitation	Uniform thickening of LV walls; no LVOT obstruction typically	Apical wall thickening; “ace-of-spades” morphology of the LV cavity; ±apical aneurysm
Hemodynamics	Dynamic LVOT obstruction present in the obstructive form; diastolic dysfunction; mid-ventricular obstruction may be present	Diastolic dysfunction; usually no LVOT obstruction.	Diastolic dysfunction; no LVOT obstruction; mid-ventricular obstruction frequent
CMR Findings	Asymmetric septal hypertrophy; LGE at RV insertion points and patchy LGE at the site of maximum hypertrophy	Symmetrical hypertrophy with variable patterns of LGE	Apical cavity systolic obliteration, loss of apical tapering; an apical aneurysm ± apical LGE and/or thrombus may be present
Clinical Manifestations	Syncope (especially if obstructive), exertional dyspnea, chest pain, palpitations, SCD	Often asymptomatic or mild symptoms; SCD risk depends on extent of fibrosis	Chest pain, palpitations; SCD less common; apical thrombosis may cause symptoms due to systemic embolization
Prognosis	Variable; high risk if obstruction or high extent of LGE present	Generally better than septal HCM unless diffuse fibrosis	Often benign course, though risk exists if apical aneurysm or fibrosis present

**Table 3:** Morphological abnormalities of the mitral valve and papillary muscles and their pathophysiological consequences.

Morphological Abnormalities of the Mitral Valve and Papillary Muscles and Their Pathophysiological Consequences	
Morphological Abnormalities	Pathophysiological Consequences
Elongated anterior (and posterior) mitral leaflet	Facilitate SAM
Anterior and basilar displacement of the anterolateral papillary muscle	Facilitate SAM and/or mid-ventricular obstruction
Abnormal muscular connection between the papillary muscle head and the anterolateral wall	
Bifid papillary muscles	
Papillary muscle abnormally inserting onto the mid-portion of the anterior mitral leaflet without intervening chordae	
Apical displacement of the papillary muscles	Usually none
Calcification of the mitral leaflets or annulus	May impede mitral valve repair if required

## Data Availability

No new data were created or analyzed in this study. Data sharing is not applicable to this article.

## Abbreviations

CMR	cardiac magnetic resonance
HCM	hypertrophic cardiomyopathy
LA	left atrium
LV	left ventricle/left ventricular
LVOT	left ventricular outflow tract
MV	mitral valve
SAM	systolic anterior motion
